# Supplementary material for: A survey-based analysis of the academic job market
Source: eLife. 2020 Jun 12;9:e54097. doi: 10.7554/eLife.54097 (PMC7360372; doi:10.7554/eLife.54097)
Supplement: Supplementary file 41. — Survey of the applicants to the tenure-track jobs. [file elife-54097-supp41.docx]

**The Job Applicant survey**

**Survey of the applicants to the tenure-track jobs**

Please answer in as much detail as you can. All responses will be treated fully anonymously.

*Required

1. Where (which country) did you apply for faculty positions?*

United States

Canada

United Kingdom

Germany

Switzerland

Australia

Singapore

Other: Short Answer---------------

1. Your field (broadly defined)*

Biomedical or life sciences

Bioengineering

Biology (other)

Chemistry

Chemical engineering

Computer Science

Mechanical engineering

Physics

Other: Short answer----------------

1. Did you apply primarily to research (R1) or teaching-focused (PU) institutions?

R1 Institutions

PUIs

Both R1 & PUIs

Other: Short answer----------------

1. Total Number of applications submitted June 2018-April 2019*

Short Answer:---------------------

1. Number of remote/offsite (e.g. phone, Skype...) interviews*

Short Answer:---------------------

1. Number of onsite interviews*

Short Answer:---------------------

1. Number of offers*

Short Answer:---------------------

1. Approximate Number of rejections

Short Answer:---------------------

1. Do you have any comments that you'd like to share? For example, how did you experience the application process? Did you use the Future PI google sheet? Did you find it helpful? If yes, why?

Long Answer:---------------------

1. Where are you currently working (country)?

Short Answer---------------

1. What's your gender?

Male

Female

Non-Binary

Prefer not to disclose

Other: Short Answer---------------

1. What is your current position?

Postdoc (or equivalent, if it is a postdoc position with a different title)

PhD student

Other: Long Answer-----------------

1. If postdoc, how many years have you been a postdoc? (TOTAL number of years, if you've had multiple appointments.)

Short answer:----------------------

1. If postdoc, is this your first postdoc position?

1st postdoc

2nd postdoc

>2nd postdoc

1. How many times have you applied for PI positions? I.e. if the 2018-2019 application cycle was the first time, please enter "1", if you also applied last cycle, enter "2", etc.

Short Answer:---------------------

1. If you have a Google Scholar account, what is your number of citations (all; since 2014), and h-index (all; since 2014)

Short Answer:---------------------

1. How many papers have you published? (ALL papers: as co-author, first author and last author. If conference abstracts count towards publications in your field, please include those as well.)

Short Answer:---------------------

1. How many first author papers have you published?

Short Answer:---------------------

1. How many papers have you published as corresponding author?

Short Answer:---------------------

1. What is your highest impact-factor publication (IF or journal)? Was this as first author, a co-author, or as corresponding author? (Suggested format for answer: journal name, author description) [e.g. eLife, first author]

Short Answer:---------------------

1. Did you have any Cell/Nature/Science publications? If yes, how many and were you first author, a co-author, or corresponding author on them? (Suggested format for answer: number, author description) [e.g. 2, first author, 1 corresponding author]

Short Answer:---------------------

If your response to previous question was "No", which journals have you prominently published in? Please name no more than 5. Please avoid ambiguous abbreviations.

1. Did you have any preprints (which were not yet accepted/published at the time of application)? If yes, how many? (Suggested format: yes/no, number)

Short Answer:---------------------

1. How many preprints have you posted throughout your career?

Short Answer:---------------------

1. Do you have any patents filed (approved or pending)? If yes, how many? (Suggested format: yes/no, number)

Short Answer:---------------------

1. Do you have any teaching experience?

No

Yes TA position(teaching assistantship)

Yes experience beyond TA

1. If "yes, beyond TA", please elaborate: (e.g. What type of experience? How many years? Was it an undergraduate or graduate course? Was the course designed by you?)

Long Answer:---------------------

1. Did you have a PhD or postdoc fellowship?

No

Yes, PhD fellowship

Yes, postdoc fellowship

Yes, both

Yes

1. Have you ever been a PI or co-PI on a grant (such as K99/K01/R01 in the US, i.e. not postdoc/training fellowships)?

No

Yes, PI

Yes, co-PI

Other: Short Answer---------------

1. If you said "Other" to previous question or would like to name the type of grant please explain.

Long Answer:---------------------

1. Did you also apply for non-faculty positions such as Industry or Government or other jobs)?

No

Yes, non-faculty positions in academia

Yes, positions outside of academia

1. If you said "Other" to the previous question or would like to explain your answer please elaborate. Long Answer:---------------------
2. Do you have any comments? For example, was any aspect of your career particularly helpful/an obstacle when applying (preprints, grants etc...)?

Long Answer:---------------------
