# Supplementary material for: A survey-based analysis of the academic job market
Source: eLife. 2020 Jun 12;9:e54097. doi: 10.7554/eLife.54097 (PMC7360372; doi:10.7554/eLife.54097)
Supplement: Supplementary file 42. — Survey of faculty members involved in tenure-track searches. [file elife-54097-supp42.docx]

**The Search Committee survey**

**Survey of faculty members involved in tenure-track searches**

Please answer in as much detail as you can. All responses will be treated fully anonymously.

*Required

1. How would you broadly define the field of the search(es) you've been involved in? *

Biological and/or biomedical/life sciences

Engineering

Computer Science

Mathematics

Physics

Chemistry

Psychology

Social Sciences

Other

1. In what country are you based at? Short Answer:---------------------
2. How would you describe your institution? *

PUI (Primarily Undergraduate Institution)

D/PU: Doctoral/Professional Universities

R1: Doctoral Universities

R2: Doctoral Universities

Independent research institute

Prefer not to disclose

Other: Short Answer---------------

1. Approximately how many applicants for a posted position do you get? *

1-19

20-49

50-99

100-199

200+

Prefer not to disclose

1. Approximately how many applicants make it through the first round of cuts? *

1-19

20-39

40-59

60+

Prefer not to disclose

1. Approximately how many applicants are invited for off-site interview (Skype/phone)? *

Short Answer---------------

1. Approximately how many applicants are invited for on-site interview ? *

Short Answer---------------

1. Approximately how many offers does your committee make per job posting? *

0-1

2-3

4+

Prefer not to disclose

1. Approximately how many openings has your department had in the last five years? *

0-1

2-3

4-5

6+

1. Does your committee weigh Cell, Science, or Nature papers above papers in other journals? *

Not at all Heavily

1 2 3 4 5

1. To what extent does journal impact factor explicitly weigh in to the selection process (e.g. does the word ‘impact factor’ come up in discussions around applicants)? *

Not at all Heavily

1 2 3 4 5

1. To what extent does good mentorship in the candidate's postdoctoral/graduate student lab explicitly weigh on selection process (e.g. "This candidate's mentor is known to produce good trainees")? *

Not at all Heavily

1 2 3 4 5

1. To what extent does the research proposal weigh on the selection process (e.g. "This candidate's research statement is incredibly compelling!")? *

Not at all Heavily

1 2 3 4 5

1. Does your committee look favorably upon preprints? *

Yes (preprints are appreciated and considered a demonstration of productivity)

No (preprints are largely ignored)

Other: Short Answer---------------

1. How heavily does the committee weigh graduate student fellowships or awards (e.g. NSF GRF, NIH F30, etc.)? *

Not at all Heavily

1 2 3 4 5

1. How heavily does the committee weigh non-transitional postdoctoral fellowships or awards (e.g. NIH F32, AHA etc.)? *

Not at all Heavily

1 2 3 4 5

1. How heavily does the committee weigh transition awards as a positive factor (i.e. K99/R00 award, Burroughs Wellcome Career Award, or some award that provides the applicant with money as a new faculty member)? *

Not at all Heavily

1 2 3 4 5

1. How heavily does the committee weigh prior teaching experience? *

Not at all Heavily

1 2 3 4 5

1. What is your perception of the job market for tenure track faculty as someone involved in the search process (please tick all that are true). *

Easy to identify good candidates from applications

Hard to identify good candidates from applications

Too many good applicants

Too few good applicants

Applicants lack sufficient teaching experience

Candidates surpass expectations during interviews

Candidates fall below expectations during interviews

The market has changed a lot since I applied for a faculty position

Other: Long Answer---------------

1. What information do you wish more candidates knew when they submit their application?

Long Answer:---------------------

1. How long have you been involved in academic search committees? *

1 - 4 years

5 - 10 years

11 - 19 years

20 - 29 year

30+ years

Prefer not to disclose

1. Have you noticed any changes in the search process since the first search you were involved in?

Long Answer:---------------------

1. Do you have any other comments or thoughts about the state of hiring for tenure track positions?

Long Answer:---------------------
