# Supplementary material for: A survey-based analysis of the academic job market
Source: eLife. 2020 Jun 12;9:e54097. doi: 10.7554/eLife.54097 (PMC7360372; doi:10.7554/eLife.54097)
Supplement: Transparent reporting form [file elife-54097-transrepform.docx]

***eLife’s* transparent reporting form**

We encourage authors to provide detailed information *within their submission* to facilitate the interpretation and replication of experiments. Authors can upload supporting documentation to indicate the use of appropriate reporting guidelines for health-related research (see [EQUATOR Network](http://www.equator-network.org/%20)), life science research (see the [BioSharing Information Resource](https://biosharing.org/)), or the [ARRIVE guidelines](http://www.plosbiology.org/article/info:doi/10.1371/journal.pbio.1000412) for reporting work involving animal research. Where applicable, authors should refer to any relevant reporting standards documents in this form.

If you have any questions, please consult our Journal Policies and/or contact us: [editorial@elifesciences.org](mailto:editorial@elifesciences.org).

**Sample-size estimation**

- You should state whether an appropriate sample size was computed when the study was being designed
- You should state the statistical method of sample size computation and any required assumptions
- If no explicit power analysis was used, you should describe how you decided what sample (replicate) size (number) to use

Please outline where this information can be found within the submission (e.g., sections or figure legends), or explain why this information doesn’t apply to your submission:

An appropriate sample size was not strictly computed ahead of study design, but it was known that approximately 100 individuals were actively using an anonymous google sheet through Future PI slack, where the idea for this study originated. We aimed for ~100 response, we received 322 over the course of 6 weeks. Some responses were filtered out of analysis as described in our materials and methods.

**Replicates**

- You should report how often each experiment was performed
- You should include a definition of biological versus technical replication
- The data obtained should be provided and sufficient information should be provided to indicate the number of independent biological and/or technical replicates
- If you encountered any outliers, you should describe how these were handled
- Criteria for exclusion/inclusion of data should be clearly stated
- High-throughput sequence data should be uploaded before submission, with a private link for reviewers provided (these are available from both GEO and ArrayExpress)

Please outline where this information can be found within the submission (e.g., sections or figure legends), or explain why this information doesn’t apply to your submission:

This study contains no replicates. 5 overall outliers were filtered out in which answers were not interpretable or did not appear to answer the correct questions. See “data analysis” in materials and methods.

**Statistical reporting**

- Statistical analysis methods should be described and justified
- Raw data should be presented in figures whenever informative to do so (typically when N per group is less than 10)
- For each experiment, you should identify the statistical tests used, exact values of N, definitions of center, methods of multiple test correction, and dispersion and precision measures (e.g., mean, median, SD, SEM, confidence intervals; and, for the major substantive results, a measure of effect size (e.g., Pearson's r, Cohen's d)
- Report exact p-values wherever possible alongside the summary statistics and 95% confidence intervals. These should be reported for all key questions and not only when the p-value is less than 0.05.

Please outline where this information can be found within the submission (e.g., sections or figure legends), or explain why this information doesn’t apply to your submission:

As per editor’s request, some of this information was taken out of the main text and moved largely to the relevant figure captions. All n values and p values are reported on each figure panel, in each figure legend, and repeated in summary in table S7. Table S7 also includes a description of each statistical test used. Table S22 describes the logistic regression analysis in detail as well. Statistical analysis methods are described and justified in “data analysis” in our materials and methods

(For large datasets, or papers with a very large number of statistical tests, you may upload a single table file with tests, Ns, etc., with reference to sections in the manuscript.)

**Group allocation**

- Indicate how samples were allocated into experimental groups (in the case of clinical studies, please specify allocation to treatment method); if randomization was used, please also state if restricted randomization was applied
- Indicate if masking was used during group allocation, data collection and/or data analysis

Please outline where this information can be found within the submission (e.g., sections or figure legends), or explain why this information doesn’t apply to your submission:

As per editor’s request, an explanation of n values is now included on page 7. It reads:

*“A total of 322 early career researchers answered survey questions regarding their experience on the academic job market in the 2018-2019 application cycle and data from 317 of these responses were used for analysis based on simple quality filters (see methods and supplemental materials). As all questions were optional, these 317 responses represent the maximum number in our analyses; in cases where respondent chose not to answer the question, we analyzed only the applicant subset with responses and list the number of responses used for each analysis in the appropriate figures and tables.”*

**Additional data files (“source data”)**

- We encourage you to upload relevant additional data files, such as numerical data that are represented as a graph in a figure, or as a summary table
- Where provided, these should be in the most useful format, and they can be uploaded as “Source data” files linked to a main figure or table
- Include model definition files including the full list of parameters used
- Include code used for data analysis (e.g., R, MatLab)
- Avoid stating that data files are “available upon request”

Please indicate the figures or tables for which source data files have been provided:

A description of “data availability” is included in our materials and methods section. It states: “The authors confirm that, for approved reasons, access restrictions apply to the data underlying the findings. Raw data underlying this study cannot be made publicly available in order to safeguard participant anonymity and that of their organizations. Ethical approval for the project was granted on the basis that only aggregated data is provided (as has been provided in the supplementary tables) (with appropriate anonymization) as part of this publication.”

Data analysis via R utilized widely available tools and is described thoroughly in our “data analysis” section of materials and methods.
